# Medical and Physical Intelligence

**Published:** 1822-11

**Authors:** 


### MEDICAL AND PHYSICAL INTELLIGENCE.

### 1. *Small-Pox after Vaccination.*

M. TOURET, aged nineteen, a student of medicine in Paris, whose father was long dean of the Faculty of Medicine, and a nephew of Desgenettes, was vaccinated in his infancy by Husson (the Jenner of France): the development of the virus followed its usual course, and was in all respects satisfactory. Last January, M. Touret was attacked with small-pox of the coherent kind, the eruption having been preceded by fever, accompanied with delirium, for several days. The case was well marked, and attracted considerable notice, having been attended by Fouquier and Desgenettes, and visited by others. One of the conclusions drawn from it is, "that a very rare event, and which has perhaps never been well established before the present circumstance, does not destroy the importance and general truth of the preservative influence of vaccination." In the conclusion we quite agree; but to tell us that this observation of M. Pelletan is the first case of small-pox after vaccination, "*bien constaté*," shows extreme ignorance or extreme prejudice: there are so many hundreds of cases, as well marked as the present, on record, that no person in this country would have thought of making such a remark.

### 2. *Preservative Power of Vaccination.*

The Central Commission of Vaccination in France have lately published a striking instance of this. The mother of four children, dwelling in a hamlet consisting of sixty houses, near Cherbourg, had two of them vaccinated, and at the end of eight days communicated the disease to the others herself. Small-pox soon after broke out in the hamlet, in many instances proving fatal, and *all* the children were attacked by it, with the exception of those who had been vaccinated. Some women, who had ridiculed the precautions of the mother, piqued to see the children escape, had the wickedness to entice them to their houses several times,—to rub their faces with small-pox matter, and to induce them to eat with their children labouring under the disease. All these attempts proved unavailing; and the women, convinced too late of the efficacy of vaccination, testified their regret at not having followed the example of their neighbour.

### 3. *Hardening of the Brain.*

PINEL (the son,) lately read to the Institute a memoir on Hardening of the Brain. He relates four cases of epilepsy, palsy, and idiocy, in which he has found this organic lesion: in all these it had its seat in the medullary substance, never extending to the cineritious; its appearance is that of a fibrous substance, resembling in many cases hardened albumen.

### 4. *Prussic Acid and Oil of Croton.*

Dr. DARWALL, in his Report of the Diseases of Birmingham, writes as follows:—"The disease of most frequent occurrence, next to dyspepsia, was phthisis pulmonalis. This was certainly, in many instances, to be referred to the employments carried on in the town, acting probably upon constitutions predisposed. In some of these cases, great relief was afforded by enjoining absence from labour. In a button burnisher, in whom there was evidently an increased determination of blood to the part, the symptoms several times disappeared under the use of digitalis, but as often returned upon resuming his employment. For this disorder, also, the prussic acid has been lauded. I can only say that, after numerous and very attentive trials, I have not seen the slightest relief afforded by it, while many patients are unable to take the smallest dose from its occasioning nausea and vomiting. The only instance in which this dangerous medicine seemed really useful was a disease of the heart, accompanied with great pain, palpitation, and copious expectoration. It is but right to add, however, that all these symptoms had raged, and disappeared in a similar manner, before this fashionable medicine was in vogue."

The same writer says,—"The oil of croton I have tried very extensively, and it is certainly a valuable addition to medicine. It has generally produced nausea and vomiting, but not so frequently griping. One circumstance attends its operation, which I have not yet seen noticed,—viz. the very little constitutional derangement which it leaves. After the operation of other drastic purgatives, a feverishness and loss of appetite remain for a considerable time, and the catharsis from elaterium is frequently alarming. But I have seen none of these consequences after the croton oil, which, however violently it may operate upon the bowels, has not, in any instance in which I have given it, (and I have given it in upwards of forty,) left the slightest disorder or uneasiness. In other respects, my experience accurately corresponds with the accounts which have been given of it by other writers.—*(Edinb. Med. and Surg. Journal.)*

### 5. *Bezoards voided by a Female.*

M. Henri Bracconot has recently examined some of these calculi, sent to him by Dr. Champion, a physician at Bar-le-Duc, *(Annales de Chimie*, tome xx.) Bezoards are calculi of a peculiar nature, found occasionally in the stomach and intestines of herbiferous animals, and to which a considerable price and marvellous properties have been sometimes attached. Some of these were sent as presents into France by the King of Persia, and an account of them published by Berthollet: they very much resembled those now described.—A woman, aged thirty-six, unmarried, of cachectic appearance, and menstruating irregularly, vomited some blood every day: this blood frequently contained the above-mentioned concretions, which are tubercular on the surface, of the shape of almonds, the size of small hazel-nuts, and of a brownish-red colour. Internally, they are of a yellowish white, appear to consist of brilliant chrystalline grains, but without concentric laminæ. Their texture is generally close, but sometimes cellular, like the part of bones which contains the marrow: they may be cut with a knife like wood, which they also resemble in appearance. At one end there is a depression, frequently filled with blood, and communicating with a tube which runs through their whole length. None have a distinct nucleus; two are hollow within; the specific gravity exceeds that of water. These bezoards are inflammable, but do not emit, during combustion, the fœtid odour which characterizes animal matters; and, according to the opinion of M. Braconnot, they could only be formed by a liquid in which small particles of a ligneous nature, in consolidating under the form of crystalline grains, become united by adhesive attraction, and so constitute these masses, having the mixed characters of wood and stone, *(masses ligneuses lapideformes.)*

### 6. *Tartar Emetic.*

The following is an extract of a letter from Mr. Bush, of Frome:—"In the Journal for September, I was gratified to see the Editors express their approbation of the use of this medicine in Hernia Humoralis. It is stated that 'Mr. Jefferys very candidly ascribes to Dr. Balfour the merit of having directed the attention to it as a powerful sedative.' On a broad scale, Dr. Balfour is, I believe, entitled to this remark; but tartrite of antimony had been long used, with the same views, by very many practitioners, before Dr. B. published on this subject; and in this Journal (vide vol. xxxvi.) I called the attention to it as a remedy for hernia humoralis, capable of arresting inflammatory action in a few days. Subsequent opportunities have uniformly confirmed my remarks made on that occasion. I may be accused of vanity,—but my paper on that subject is, as far as I know, the first in which emetic tartar is recommended as capable of subduing the disease, without local or general bleeding."

### 6. *Magnesia an Antidote to Arsenic.*

Our readers will probably recollect that Mr. HUME published in the Number of this Journal for November, 1821, a Case of Poisoning from Arsenic successfully treated by the Exhibition of Carbonate of Magnesia. We are requested by that gentleman to announce, that a case confirming his opinion of the efficacy of that medicine under similar circumstances, has lately been transmitted to him from the country, and the particulars of which he has promised to communicate to us in the ensuing Number.

### 7. *Ovarian Dropsy cured by Operation.*

Dr. N. SMITH, professor of physic and surgery in Yale College, Connecticut, removed the sac of an encysted dropsy from the abdomen of a woman, aged thirty-three, whose general health was not much impaired. We are told that the tumor was "large," and movable to a considerable extent. The operation, which was performed on the 5th of July, 1821, is thus described:

The patient being placed on a bed with her head and shoulders somewhat raised, an assistant pushed up the tumor to the middle of the abdomen, and held it there. Dr. Smith then commenced an incision about an inch below the umbilicus, directly in the linea alba, and extended it downwards three inches. The peritoneum was then divided the whole extent of the external incision. The tumor, now exposed to view, was punctured; a canula was introduced, and seven pints of a dark-coloured ropy fluid were discharged into a vessel. About one pint was lost, so that the whole was about eight pounds. Previous to puncturing the tumor, by introducing the finger by the side of it, Dr. S. ascertained that it adhered for some extent to the parietes of the abdomen, on the right side, between the spine of the ilium and the false ribs. After evacuating the fluid, he drew out the sac, which brought out with it, and adhering to it, a considerable portion of omentum. This was separated from the sac by the knife; and two arteries, which he feared might bleed, were tied with leather ligatures, and the omentum was returned. By continuing to pull out the sac, the ovarian ligament was brought out; it was cut off; two small arteries were secured as before, and the ligament returned. He then endeavoured to separate the sac from its adhesions to the parietes of the abdomen, which occupied a space about two inches square. This was effected by a slight touch of the knife at the anterior part of the adhesion, and by the use of the fingers. The sac then came out whole, excepting where the puncture had been made, and weighed between two and four ounces.

The incision was then closed with adhesive plaster, and a bandage applied round the abdomen. No unfavourable symptoms occurred after the operation. In three weeks the patient was able to sit up and walk, and has since perfectly recovered.

Dr. S. was induced to undertake the operation from the following considerations:—The patient, although her health was not greatly injured, was sensibly affected by the disease. She was quite certain that the increase of the tumor in a given time was augmented; and probably, at no very distant period, it would destroy her. He had also had an opportunity to dissect the body of a patient who had died of ovarian dropsy, after having been tapped seven times. In this case, the sac was found to be the right ovarium, which filled the whole abdomen, but adhered to no part except the proper ligament, which was not larger than the finger. He had seen two other ovarian sacs, which were taken from patients after death: they had been tapped several times, and the sacs were equally unattached except to their own ligaments. Thence he inferred that, in a case of ovarian dropsy, while the tumor remained movable, it might be removed with a prospect of success; and the event has justified his expectations.

### 8. *Pulvis Antimonialis.*

Our readers may remember that Dr. Elliotson, in a little pamphlet of considerable interest, detailed, among other matters, several cases in which large doses of the pulvis antimonialis had been taken without any sensible effect. This circumstance induced Mr. Richard Phillips to make some experiments, by which he has been led to the same conclusion as M. Chenevix,—viz. that antimonial powder consists of oxide of antimony and bone earth.

For the purpose of determining the quantities of the peroxide of antimony and phosphate of lime contained in the pulvis antimonialis from Apothecaries' Hall, two hundred grains were boiled for a long time in about three ounces of strong muriatic acid: seventy grains of peroxide of antimony were left undissolved, and consequently the powder consists of

| | |
|---|---|
| Peroxide of antimony | 35 |
| Phosphate of lime | 65 |
| | 100 |

Mr. Phillips now procured some antimonial powder from another source, but of respectability equal to that above mentioned: he could discover no difference in their appearance, but it was heavier than that from the Hall, in the proportion of about 100 to 85. With this powder, he repeated experiments similar to those just detailed, and with similar results: it was a mere mixture of peroxide of antimony and phosphate of lime, containing, however, rather more of the oxide. It consisted of

| | |
|---|---|
| Peroxide of antimony | 38 |
| Phosphate of lime | 62 |
| | 100 |

The experiments now detailed will, Mr. P. thinks, sufficiently account for the inertness of the pulvis antimonialis: it can only be regarded as a mixture of phosphate of lime with the old diaphoretic antimony; a preparation of antimony now quite out of use, on account of its deficiency of power, and which is not likely to be increased by admixture with phosphate of lime.

That the antimony should be thus converted into peroxide will be readily conceived, when it is remembered how slowly metallic sulphurets part with the last portions of sulphur, and animal matter with all the carbon it contains.

M. Chenevix has proposed to precipitate together protoxide of antimony and phosphate of lime; and, provided a mixture of protoxide of antimony and phosphate of lime possessed any efficacy which does not equally belong to oxide of antimony mixed with any other inert substance, his process is unquestionably a good one. It is, however, worthy of the consideration of medical men, whether tartarized antimony in small doses may not be advantageously substituted for every other antimonial preparation. It possesses the great advantages of being readily procured of certain and uniform power.—*(Annals of Philosophy.)*

### 9. *Consumption.*

Considerable attention has been excited, in America, to a species of consumption depending on an elongation of the uvula, causing irritation; and which is relieved by cutting a portion of it off.

### 10. *College of Surgeons.*

At the last annual election at the Royal College of Surgeons, th following officers were chosen. It will be perceived that, in conformity with the new charter which his Majesty has been graciously pleased to grant to the College, the names of Master, Governors, and Court of Assistants, are now changed to President, Vice-Presidents, and Council. His Majesty has also bestowed upon the College a further proof of his generosity and patronage, by presenting them with an elegant silver-gilt mace, which is borne before the president upon all solemn occasions.

*President.*—Sir William Blizard.

*Vice-Presidents.*—Henry Cline and William Norris.

*Council.*—Thompson Forster, John Heaviside, Sir David Dundas, Bart. Sir Everard Home, Bart. John Adair Hawkins, Francis Knight, Sir Ludford Harvey, Knt. William Lynn, John Abernethy, William Lucas, Sir A. P. Cooper, Bart. Sir Anthony Carlisle, Knt. Thomas Chevalier, John Gunning, H. Leigh Thomas, Richard C. Headington, Robert Keate, and John P. Vincent.

---

*** It is with much regret we have to announce the death of Dr. Alexander Marcet, which took place at Great Coram-street, on the 20th ult. when on his way from Scotland to Geneva. In our next Number, we trust we shall be able to give some particulars relative to this distinguished physician.

### NEW REGULATIONS RESPECTING APOTHECARIES.

### 1. *Apothecaries practising without Certificates of Qualification.*

SINCE the year 1815, when the Act of Parliament for better regulating the Practice of Apothecaries was passed, numerous informations have been sent to the Society, against persons said to be practising as apothecaries, not being qualified according to the terms of the Act.

Many of these informations have been communicated through the medium of anonymous letters, which neither stated any particulars, nor pointed out the means of procuring the evidence necessary to enable the Society to proceed effectually against the parties complained of.

Many other informations have, upon investigation, been discovered to have been given against persons who have since proved satisfactorily that they had been in practice as apothecaries previously to the 1st of August, 1815; and, in other cases, the parties who have given the informations have been unable to furnish such evidence as would warrant the Society in taking legal measures against them.

Many persons, against whom informations had been given, have been written to by the solicitor of the Society; of these, some have submitted to the examination required by the Act, and others have ceased to practise.

Since the Act passed, the Society have brought actions against four persons, and in all these cases have obtained a verdict.

The first case was that of a gentleman in Sussex, who had served a regular apprenticeship, but who had not practised on his own account previously to the 1st of August, 1815. In this instance the Society obtained a judgment by default, the defendant admitting his liability to the penalty. He has since appeared before the Court of Examiners, and, having proved his fitness and qualification to practise as an apothecary, has received the certificate directed by the Act.

In the second case, the cause was tried at Stafford. The defendant endeavoured to prove that he had been in practice on his own account previously to the 1st of August, 1815; but the judge, who tried the cause, being of opinion that his total want of medical education rendered him incapable of having ever acted as an apothecary, inasmuch as it would have been impossible for him to have complied with the fifth section of the Act, the jury decided against him. This verdict the defendant attempted to set aside, on the ground that the judge was wrong in directing the jury in the manner he had done; and the case having been argued in the Court of King's Bench, upon a rule obtained by the defendant, calling on the Society to show cause why the verdict should not be set aside, the judges unanimously decided that the judge who tried the cause had directed the jury properly, and the verdict was consequently confirmed.

The third case was tried last spring, and the defendant in this instance also endeavoured to establish his claim to practise by having been, previously to August 1, 1815, already in practice as an apothecary. It appeared, by the evidence on the trial, that the person to whom he was apprenticed died in the month of April or May, 1815, when he had served about three years and a half of his apprenticeship, and had attained only seventeen or eighteen years of age; and that, about the middle of June following, he engaged himself as an assistant to an apothecary, and received a salary for his services. In the interval which happened between the death of his master and the operation of the Act of Parliament, this person offered proofs of his having attended two or three patients on his own account; but the jury, under the direction of the chief justice of the Court of King's Bench, returned a verdict for the plaintiffs; which verdict has been since confirmed upon argument before the judges of the same court, in consequence of a rule having been obtained by the defendant, calling on the plaintiffs, as in the last case, to show cause why the verdict should not be set aside. This gentleman had been called upon, previously to any proceedings being taken against him, to submit himself to an examination, but he refused to do so. He has, since the trial, been examined, and has received a certificate of qualification.

The last case was tried at York, at the summer assizes for the present year, 1822. It was proved in this case that the defendant, at the time the Act passed, and for some time afterwards, was a journeyman wool-card maker. His defence to the action was, that, during the time he was so employed, he also practised as an apothecary. The judge, however, as well as the jury, were of opinion that a defence of this nature was quite frivolous; and, without calling on the plaintiff's counsel for a reply, a verdict was given for one penalty, the plaintiffs not wishing to press for more, although the judge distinctly stated, that the defendant was liable to a *separate penalty for each case* in which it could be proved that he had acted as an apothecary. Had the plaintiffs been so inclined, they could in this case, as well as in the two last, have proved seven or eight distinct acts of practice.

By the result of these trials, it is clearly established that, in order to entitle persons legally to practise as apothecaries, it is necessary they should have been actually in practice as such on their own account, with a view of obtaining their livelihood thereby, previously to, or on, the 1st of August, 1815, and not as assistants to other persons; and that a few casual instances of attending patients are not sufficient to entitle them to the exemption from the penalties directed by the Act of Parliament.

The Society of Apothecaries are fully aware of the great importance of the trust which the legislature has confided to them, and are determined to enforce the law in all cases which may come to their knowledge, at whatsoever distance from London they may occur, or with whatsoever expences the proceedings may be attended, where *sufficient evidence* can be obtained to enable them to do so effectually. They feel assured that there does not exist any just cause of complaint against them, on account of their having declined, in some instances, to act upon statements of parties, unsubstantiated by *such* proof as their legal advisers have thought indispensably necessary; although they are well

3

aware that the parties giving the information have considered the evidence and information furnished by them as fully sufficient, and such as ought to have induced the Society to commence proceedings immediately against the persons complained of.

### 2. *Visiting Apothecaries' Shops.*

We understand that the Society of Apothecaries, in compliance with the authority granted them in the "Act for better regulating the Practice of Apothecaries throughout England and Wales," sent a deputation to York in August, for the purpose of examining the shops of the apothecaries; and a similar deputation for a like purpose to Bristol, in the month of September. For the information of those whom it may concern, we subjoin an extract which relates to this point:

"That the said master, wardens, and society of apothecaries for the time being, and their successors, or any of the assistants, or any other person or persons properly qualified, as the Act directs, to be by the master and wardens nominated and assigned, not being fewer in number than two persons at the least, shall and may from time to time, and at all seasonable and convenient times in the day-time, as often as to the said master and wardens it shall seem expedient, go and enter into any shop or shops of any person or persons whatever using or exercising the art or mystery of an apothecary, in any part of England or Wales, and shall and may search, survey, prove, and determine if the medicines, simple or compound, wares, drugs, or any thing or things whatsoever therein contained, and belonging to the art or mystery of apothecaries aforesaid, be wholesome, meet, and fit for the cure, health, and ease of his Majesty's subjects; and all and every such medicines, wares, drugs, and all other things belonging to the aforesaid art, which they shall find false, unlawful, deceitful, stale, unwholesome, corrupt, pernicious, or hurtful, shall and may burn or otherwise destroy; and also shall and may report to the master, wardens, and assistants of the said Society, the name or names of such person or persons as shall be found to have the same in their possession; and the said master, wardens, and assistants shall and may impose and levy the following fines and penalties upon each and every person whose name shall be so reported to them, as hereinafter mentioned: for the first offence the sum of five pounds, for the second offence the sum of ten pounds, and for the third and every other offence the sum of twenty pounds.

### 3. *Articles of Apprenticeship.*

Candidates for examination (before the Court of Examiners of the Society of Apothecaries,) are requested to take notice, that in future the production of their articles of apprenticeship, where such articles are in existence, will be considered indispensably requisite to examination; but, in any case where such articles shall have been lost, or from any other cause may not be capable of being produced, it is expected that the candidate shall bring forward very strong testimony to prove that he has served such an apprenticeship to an apothecary, as the Act of Parliament directs.

### 4. *Certificates signed by Physicians.*

After the 1st of January, 1823, no testimonial of attendance on lectures on the Principles and Practice of Medicine, delivered in London or within seven miles thereof, will render a candidate eligible for examination as apothecary, unless such lectures were given, and the testimonial is signed, by a fellow, candidate, or licentiate of the Royal College of Physicians.

---

### METEOROLOGICAL JOURNAL.

By Messrs. William Harris and Co. 50, Holborn, London.

*From September* 20, 1822, *to October* 19, 1822.

| | MOON. | Rain gauge | THERM. | | | BAROM. | | DE LUC'S HYG. | | WIND. | | ATMOSPHERIC VARIATION. | | |
|---|---|---|---|---|---|---|---|---|---|---|---|---|---|---|
| | | | 9 A.M. | MAX. | MIN. | 9 A.M. | 10P.M. | 9 A.M. | 10P.M. | 9 A.M. | 10P.M. | 9 A.M. | 2 P.M. | 10P.M. |
| Sep. | | | | | | | | | | | | | | |
| 20 | | | 60 | 64 | 52 | 29.87 | 29.80 | 53 | 54 | E | E | Fine | | |
| 21 | | | 54 | 60 | 52 | 29.76 | 29.71 | 59 | 60 | E | E | Fine | | |
| 22 | | | 55 | 62 | 52 | 29.73 | 29.69 | 64 | 68 | E | E | Fine | | Cloud. |
| 23 | ☾ | .50 | 56 | 59 | 50 | 29.66 | 29.52 | 89 | 90 | E | SE | Rain | Rain | Sho'ry |
| 24 | | .22 | 58 | 59 | 49 | 29.22 | 29.22 | 92 | 95 | E | E | Cloudy | Sleet | Rain |
| 25 | | | 54 | 54 | 42 | 29.21 | 29.40 | 75 | 72 | NE | NE | Fine | Cloud. | |
| 26 | | | 52 | 59 | 46 | 29.55 | 29.76 | 74 | 62 | NE | NE | Fine | | |
| 27 | | | 50 | 60 | 56 | 29.98 | 30.03 | 63 | 65 | NE | ENE | Fine | | |
| 28 | | .02 | 48 | 58 | 48 | 30.04 | 30.00 | 65 | 67 | ENE | E | Rain | Cloud. | Fine |
| 29 | | | 53 | 60 | 48 | 29.90 | 29.80 | 70 | 62 | E | E | Fine | | |
| 30 | ○ | | 54 | 60 | 44 | 29.70 | 29.63 | 60 | 65 | SE | E | Fine | | |
| Oct. | | | | | | | | | | | | | | |
| 1 | | | 55 | 60 | 45 | 29.56 | 29.56 | 75 | 69 | SE | ESE | Fine | | |
| 2 | | .34 | 60 | 65 | 58 | 29.60 | 29.57 | 82 | 82 | SSE | SSE | Showery | Rain | Cloud. |
| 3 | | .18 | 61 | 66 | 52 | 29.58 | 29.57 | 88 | 76 | SE | S | SleetFog | Rain | Cloud. |
| 4 | | | 60 | 64 | 56 | 29.61 | 29.57 | 82 | 75 | SSW | WSW | Dense F. | Rain | Cl. Li. |
| 5 | | .23 | 63 | 67 | 48 | 29.65 | 29.32 | 73 | 76 | WSW | S | Fine | Fine | Rain |
| 6 | | | 54 | 59 | 50 | 29.38 | 29.54 | 68 | 59 | W | WSW | Fine | | |
| 7 | ☽ | .20 | 56 | 64 | 54 | 29.30 | 29.54 | 66 | 57 | SW | WSW | Cloudy | Cloud. | Fine |
| 8 | | .10 | 58 | 60 | 50 | 29.31 | 29.52 | 72 | 63 | WNW | W | Rain | Cloud. | Fine |
| 9 | | .40 | 57 | 62 | 52 | 29.57 | 29.51 | 70 | 72 | SW | WSW | Fine | Cloud. | Rain |
| 10 | | .36 | 54 | 57 | 43 | 29.68 | 29.80 | 68 | 67 | W | WSW | Cloudy | Fine | Rain |
| 11 | | | 50 | 54 | 44 | 29.98 | 30.00 | 68 | 66 | W | WSW | Fine | | |
| 12 | | | 52 | 60 | 58 | 29.80 | 29.54 | 72 | 72 | S | S | Fine | | Cloud. |
| 13 | | .16 | 62 | 62 | 46 | 29.32 | 29.28 | 72 | 70 | S | NE | Cloudy | Rain | Cloud. |
| 14 | | | 48 | 52 | 36 | 29.67 | 29.82 | 68 | 64 | NE | NE | Fine | | |
| 15 | ● | .14 | 42 | 54 | 46 | 29.75 | 29.51 | 66 | 68 | NNW | SSW | Fine | | Rain |
| 16 | | .20 | 50 | 51 | 48 | 29.24 | 29.18 | 86 | 82 | SE | NNE | Showery | Rain | Cloud. |
| 17 | | .29 | 50 | 50 | 40 | 29.16 | 29.31 | 94 | 78 | ENE | NE | Rain | | Sho'ry |
| 18 | | .14 | 40 | 45 | 43 | 29.50 | 29.50 | 75 | 85 | N | WNW | Fine | | Rain |
| 19 | | .21 | 50 | 52 | 52 | 29.34 | 29.34 | 78 | 79 | SW | WSW | Cloudy | Rain | Cloud. |

The quantity of rain that fell in the month of September, was 80,100ths.

